# Nomograms for Predicting Risk and Survival of Esophageal Cancer Lung Metastases: a SEER Analysis

**DOI:** 10.7150/jca.92389

**Published:** 2024-04-23

**Authors:** Wenhui He, Youzhen Yu, Ziting Yan, Na Luo, Wenwen Yang, Fanfan Li, Hongying Jin, Yimei Zhang, Xiaoli Ma, Minjie Ma

**Affiliations:** 1 Department of Thoracic Surgery, the First Hospital of Lanzhou University, Lanzhou 730000, Gansu Province, China.; 2 School of Nursing, Gansu University of Traditional Chinese Medicine, Lanzhou730000, Gansu Province, China.; 3 The First Clinical Medical College, Lanzhou University, Lanzhou 730000, Gansu Province, China.; 4 Gansu Province International Cooperation Base for Research and Application of Key technology of Thoracic Surgery, The First Hospital of Lanzhou University, Lanzhou, 730000, Gansu Province, China.

**Keywords:** SEER, esophageal cancer, lung metastasis, nomogram, Cox regression, logistic regression

## Abstract

**Background:** The overall survival rate is notably low for esophageal cancer patients with lung metastases (LM), presenting significant challenges in their treatment.

**Methods:** Through the Surveillance, Epidemiology, and End Results (SEER) program, individuals diagnosed with esophageal cancer between 2010 and 2015 were enrolled. Based on whether esophageal cancer metastasized to the lungs, we used propensity score matching (PSM) to balance correlated variables. Propensity score matching was a critical step in our study that helped to minimize the impact of possible confounders on the study results. We balanced variables related to lung metastases using the PSM method to ensure more accurate comparisons between the study and control groups. Specifically, we performed PSM in the following steps. First, we performed a univariate logistic regression analysis to screen for variables associated with lung metastasis. For each patient, we calculated their propensity scores using a logistic regression model, taking into account several factors, including gender, T-stage, N-stage, surgical history, radiotherapy history, chemotherapy history, and bone/brain/liver metastases. We used a 1:1 matching ratio based on the propensity score to ensure more balanced baseline characteristics between the study and control groups after matching. After matching, we validated the balance of baseline characteristics to ensure that the effect of confounders was minimized. We used logistic regression to identify risk variables for LM, while Cox regression was used to find independent prognostic factors. We then created nomograms and assessed their accuracy using the calibration curve, receiver operating curves (ROC), and C index.

**Results:** In the post-PSM cohort, individuals diagnosed with LM experienced a median overall survival (OS) of 5.0 months (95% confidence interval [*CI*] 4.3-5.7), which was significantly lower than those without LM (*P*<0.001). LM has been associated to sex, T stage, N stage, surgery, radiation, chemotherapy, and bone/brain/liver metastases. LM survival was affected by radiation, chemotherapy, and bone/liver metastases. The nomograms' predictive power was proved using the ROC curve, C-index, and validation curve.

**Conclusion:** Patients with LM have a worse chance of surviving esophageal cancer. The nomograms can effectively predict the risk and prognosis of lung metastases from esophageal cancer.

## Introduction

As of 2020, esophageal cancer holds the 10th position globally, reporting 604,100 new cases and ranking 6th for new fatalities with 544,076 deaths 1. The primary histopathological variants of esophageal carcinoma include squamous cell carcinoma and adenocarcinoma, with squamous cell carcinoma having the highest incidence in Eastern and South-East Asia and adenocarcinoma having the highest incidence in Western and Northern Europe, Oceania, and Northern America 2. Esophageal cancer, particularly adenocarcinoma, has become more common in Western countries in recent decades 3, 4. Esophageal cancer spreads quickly after it has developed. More than half of esophageal cancers had unresectable tumors or metastases at the time of diagnosis 5. Patients with metastatic esophageal cancer have a very low 5-year survival rate, with only approximately 5% surviving five years 6, 7. CT and PET/CT are two typical approaches for detecting distant lung metastases in esophageal cancer 8-10. The high cost and invasiveness of these tests, on the other hand, raise the financial burden on patients and the risk of iatrogenic damage. As a result, identifying and assessing risk factors is important for improving the effectiveness of lung metastasis screening in patients with esophageal cancer.

The lung, second only to the liver and higher than the bone and brain, is one of the most common distant metastatic sites of esophageal cancer 11-13. Some publications about esophageal cancer distant metastasis have previously been published 14, 15. Xin Tang *et al.* 14 developed a nomogram for predicting cancer-specific survival of metastatic esophageal cancer. Shizhao Cheng *et al.* 15 created a nomogram to make a prediction on the risk and prognosis of esophageal cancer brain metastases. Jida Guo *et al.* 16 looked at esophageal cancer lung metastasis, but they didn't create a nomogram, which limited their findings. As a result, developing models to predict the prognosis and risk of esophageal cancer lung metastasis is critical. We developed two nomograms to predict the survival time of lung metastases and the risk of esophageal cancer lung metastases using the SEER data from 2010 to 2015.

## Methods

### Patients

**Figure 1:**
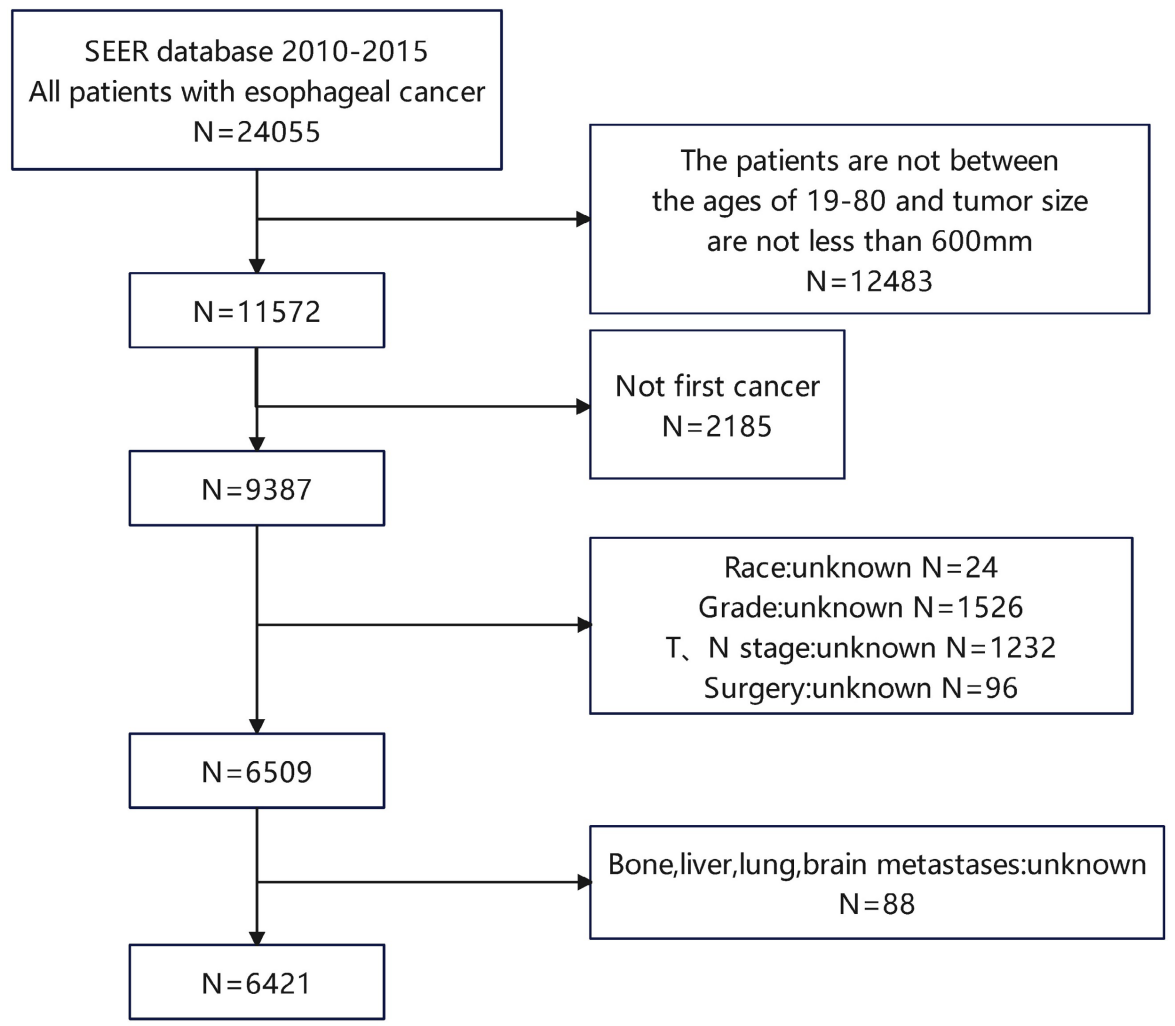
** Patient screening flowchart.** This figure contains how we screened esophageal cancer patients from the SEER database.

The retrospective study drew its data from the SEER database 18 Regs custom dataset spanning the years 1975 to 2016. Between 2010 and 2015, we screened 6421 patients with esophageal cancer, including 400 patients with LM (Figure 1). Patients who are enrolled must meet the following requirements: (1) Between the ages of 19 and 80; (2) with a tumor size of less than 600mm. The following is a summary of the exclusion criteria: (1) Not first cancer (2) Patients with stage T0 esophageal cancer (3) Patients with insufficient information (4) Individuals detected through post-mortem examinations. The SEER database is used to extract race, gender, year of diagnosis, primary site, T stage, N stage, radiation history, chemotherapy history, surgical history, tumor size, histological type, age, and bone/brain/liver/lung metastases, as well as other follow-up information. This study evaluates the survival time as the duration from the date of diagnosis to the date of either all-cause death or the last follow-up, commonly known as OS. The AJCC 7th edition was used for TNM staging. Because the SEER database is an open database, no institutional review board permission was required for this study.

### Statistical analysis and optimal cutoffs

We used Fisher's exact test or chi-square to compare differences in categorical variables. We incorporated the factors of *P*<0.05 in univariate logistic regression into multivariate regression and created a nomogram for predicting the risk of LM. By year of diagnosis, we divide 400 patients with LM into training (2012-2015, n=268) and internal validation (2010-2011, n=132) groups. We included the factor of *P*<0.10 in univariate cox regression into multivariate regression. We created a prognostic chart to forecast LM survival, and employed C-index, ROC curves, and calibration curves to confirm its accuracy. The Kaplan-Meier curve was used to assess the variance in survival duration between patients with LM and those without LM. To find the best cutoffs for tumor size and age, we used the x-tile v3.6.1 (Yale University) program 17. To balance differences in other characteristics between LM and non-LM patients, a 1:1 PSM was done in SPSS v26.0 (SPSS Inc). Statistical analyses were conducted utilizing GraphPad Prism v8.0.2 (GraphPad Software, Inc.), SPSS v26.0 (SPSS Inc.), and R software v4.1.3 (https://www.r-project.org/). A significance level of *P* < 0.05 was considered to indicate statistical significance.

## Results

### Features of esophageal cancer patients

In this retrospective investigation, we included all 6421 patients diagnosed with esophageal cancer between 2010 and 2015, with 6.2% (n=400) having lung metastases, 9.7% (n=620) having liver metastases, 5.2% (n=336) having bone metastases, 1.3% (n=86) having brain metastases, 82.1% (n=5275) were male, 84.2% (n=5408) were white, 66.4% (n=4268) were in the lower third of the esophagus, 62.4% (n=4012) were adenocarcinoma, 42.9% (n=2756) underwent surgery, 67.8% (n=4358) received radiation therapy, and 75.2% (n=4834) received chemotherapy. T3 (n=2 992, 46.5%) and N1 (n=2866, 44.6%) were the most prevalent T and N phases, respectively. In both the pre-PSM and post-PSM cohorts, we scrutinized the characteristics of the patients, as indicated in Table 1, and found no statistically significant differences in most variables in the post-PSM cohort. As a result, PSM reduces the interference of other elements.

### Survival analysis of esophageal cancer lung metastases

**Figure 2:**
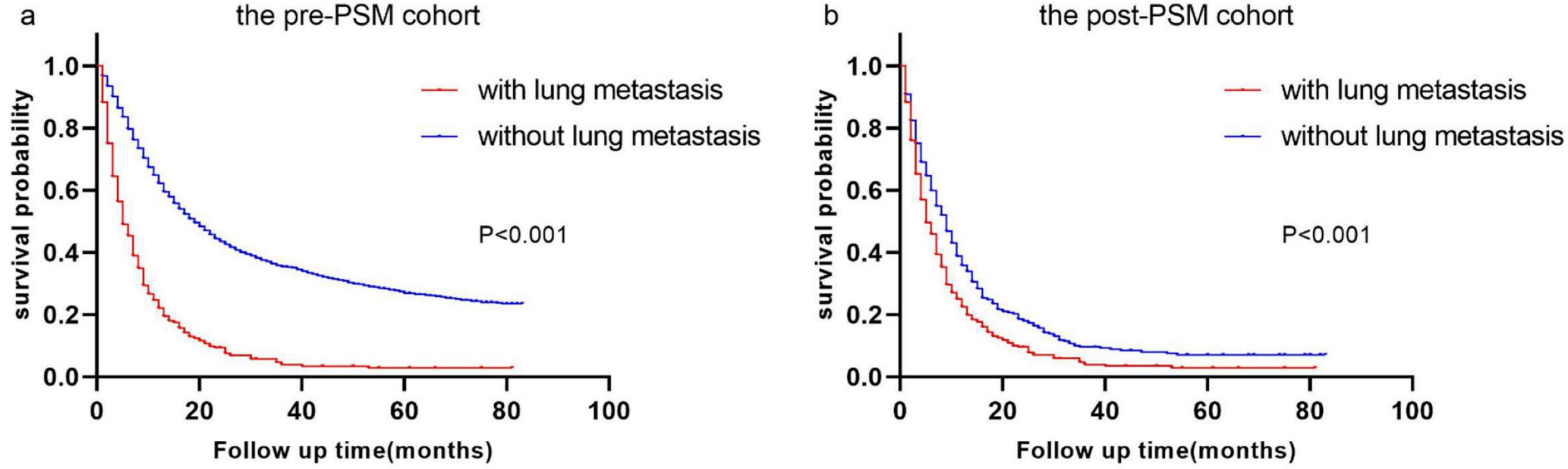
** Kaplan-Meier curves of the pre- and post-PSM cohorts.** Kaplan‒Meier curves of **(a)** the pre-PSM cohort and **(b)** the post-PSM cohort.

Before and after PSM, the cohorts had median follow-up times of 15.0 months (IQR 7.0-30.0 months) and 6 months (IQR 2.0-13.0 months), respectively. The differences in these characteristics were essentially balanced (*P* > 0.05) when 393 esophageal cancer patients with LM were matched with 393 esophageal cancer patients without LM. During the pre- and post-PSM cohorts' follow-up periods, 4308 (67.0%) and 721 (91.7%) cases died, respectively. Esophageal cancer patients with LM and those without LM had a median OS of 5.0 (95 % *CI*: 4.3-5.7) months and 18.0 (95 % *CI*: 17.1-18.9) months, respectively, in the pre-PSM cohort (Figure 2 a). Patients with esophageal cancer LM and those without lung metastases had median OS of 5.0 (95%*CI*: 4.3-5.7) months and 8.0 (95%*CI*: 6.8-9.2) months, respectively, in the post-PSM cohort, and they were statistically significantly different (Figure 2 b).

### The diagnosis possibility of LM in esophageal cancer patients

**Figure 3:**
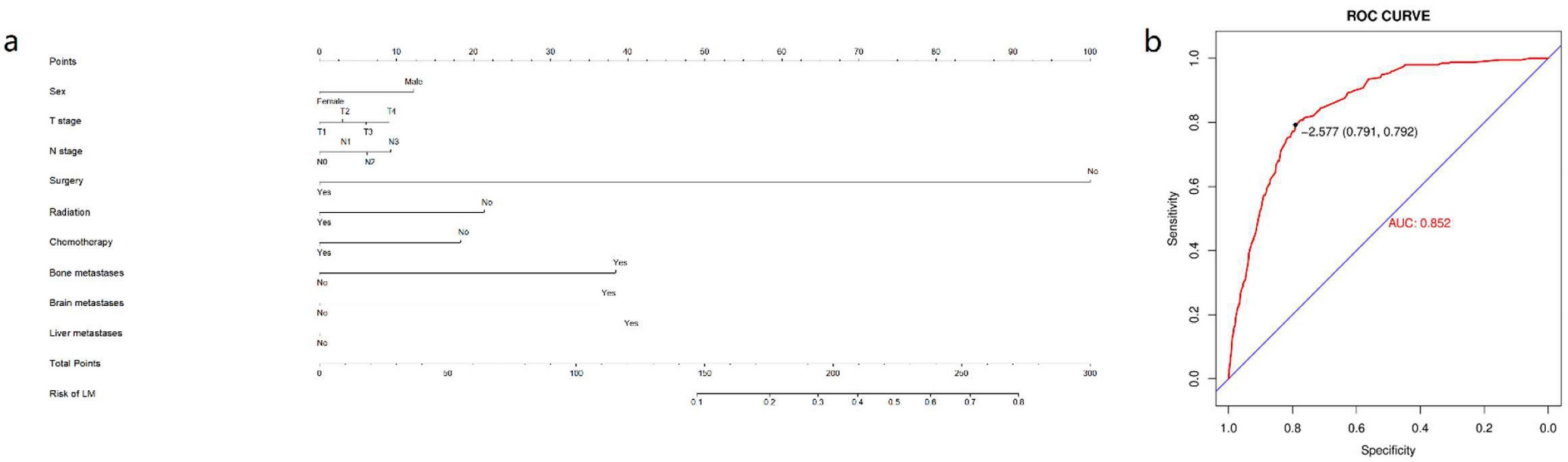
** Nomogram for predicting the diagnosis possibility of LM in esophageal cancer patients.** Nomogram for predicting the diagnosis possibility of LM in esophageal cancer patients **(a)** and ROC curve of this nomogram **(b)**.

Race, T stage, N stage, sex, primary site, grade, surgery history, histologic type, radiation history, chemotherapy history, tumor size, and bone/brain/liver metastases were all included in multivariate analysis using univariate logistic regression. P values for sex, T stage, N stage, surgical history, radiation history, chemotherapy history, and bone/brain/liver metastases remained less than 0.05 in multivariate logistic regression. Refer to Table 2 for more information. We developed a nomogram based on the foregoing findings that can reliably predict the diagnostic likelihood of LM in patients with esophageal cancer (Figure 3 a). The nomogram's C-index is 0.852, and the ROC curve is given in Figure 3 b, indicating that the model has strong predictive power.

### Independent prognostic factors in esophageal cancer LM patients

The clinical characteristics of the cohort for building the nomogram and the cohort for validating the nomogram were shown in Table 3. Fisher's exact test or Chi-square revealed that most variables across the two cohorts were very similar (*P*≥0.05). Race, T stage, grade, histological type, radiation, surgery, chemotherapy, and bone/brain/liver metastases were all included in the multivariate analysis in the training cohort, with *P*<0.10 in univariate cox regression. (see Table 4) In esophageal cancer patients with lung metastases, multivariate analysis revealed that radiation, chemotherapy, and bone/liver metastases were independent predictive variables for OS (*P*<0.05) (see Table 4).

### Construction of a prognostic nomogram and validation

**Figure 4:**
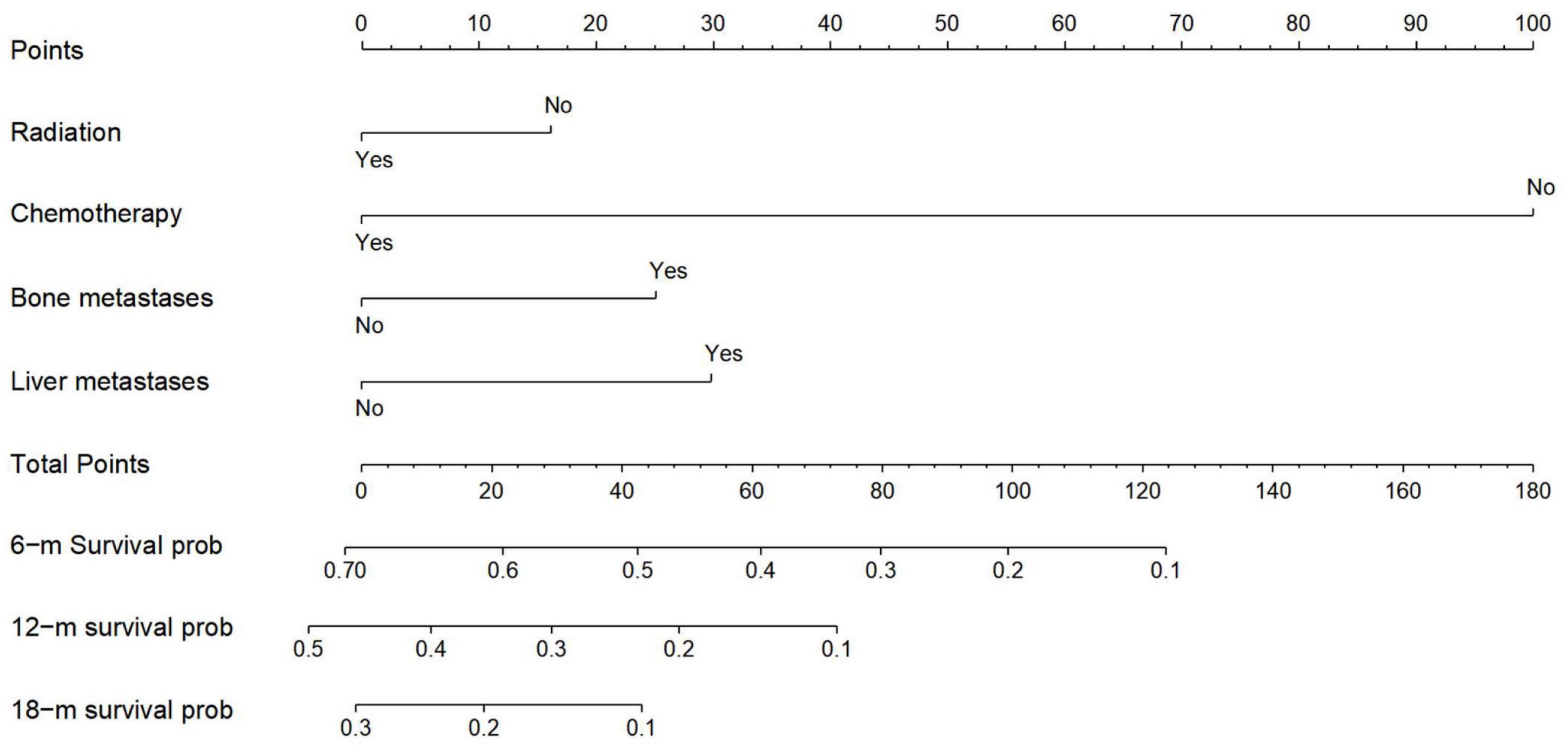
** Nomogram for predicting the overall survival of patients with esophageal cancer lung metastases.** Nomogram for predicting the overall survival of patients with esophageal cancer lung metastases. From this nomogram, the overall probability of survival at 6 months, 12 months, and 18 months can be determined.

**Figure 5:**
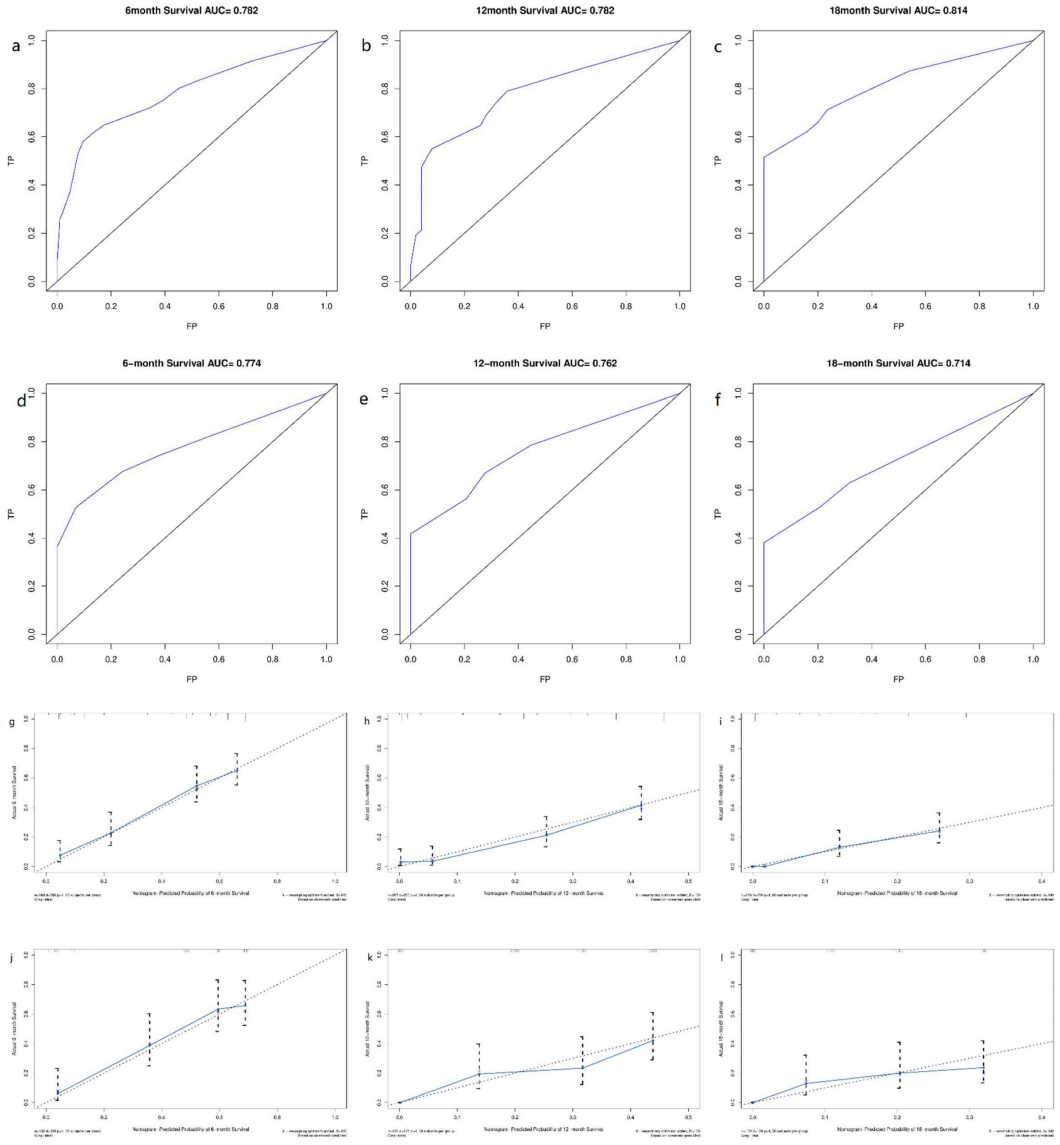
** ROC curves and calibration curves of the nomogram.** ROC curves of the nomogram to predict 6-, 12- and 18-month OS in the training cohort **(a-c)** and the internal validation cohort **(d-f)**. Calibration curves of 6-, 12- and 18-month OS for esophageal cancer lung metastases patients in the training cohort **(g-i)** and the internal validation cohort **(j-l)**. The closer the dashed line and the blue solid line are, the more accurate the model is. FP: false positive rate; TP: true positive rate.

**Figure 6:**
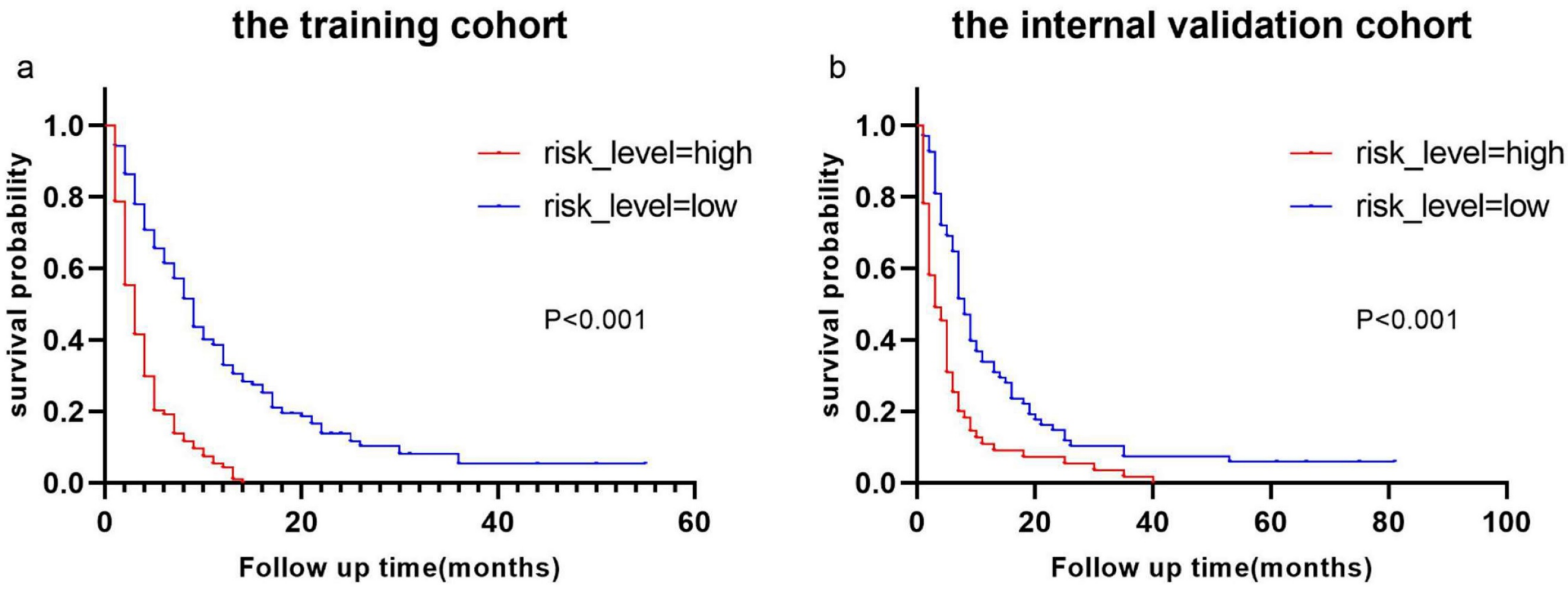
Kaplan-Meier curves of OS for patients with different risks of esophageal cancer lung metastases in low-risk and high-risk cohorts. Kaplan-Meier curves of OS for patients with different risks of esophageal cancer lung metastases in the training cohort **(a)** and the internal validation cohort **(b)**.

Derived from the results of multivariate Cox regression analysis, we created a nomogram that can forecast the prognosis of esophageal cancer lung metastases (Figure 4). The AUC of 6-, 12-, and 18-month OS in the training cohort were 0.782, 0.782, and 0.814, respectively (Figures 5 a-c). They are 0.774, 0.762, and 0.714 in the internal validation cohort, respectively (Figures 5 d-f). The training and validation cohorts had C-indices of 0.7226 (95 % *CI*: 0.6893-0.7558) and 0.7228 (95 % *CI*: 0.6737-0.7719), respectively. The C indices are greater than 0.7, indicating that this prediction model is accurate. Both the training cohort (Figures 5 g-i) and the validation cohort (Figures 5 j-l) calibration curves demonstrated a good correlation between actual and projected survival. The training and internal validation Kaplan-Meier curves Patients in different risk classes exhibited statistically significant differences in survival time, as revealed by the analysis (Figures 6 a, b).

## Discussion

The lung is one of the most common metastatic sites for esophageal cancer 11, 12, 18 and in our study, 6.2% (n=400) of esophageal cancer patients developed lung metastasis, which was second only to liver metastasis. Esophageal cancer lung metastasis has a very poor prognosis. Despite the fact that 63.0% (n=252) of esophageal cancer patients with lung metastases in this study received chemotherapy and 46.5 % (n=186) received radiotherapy, individuals with lung metastases from esophageal cancer exhibited a median survival time of merely 5 months, demonstrating the importance of early detection and treatment in improving patients' quality of life. Several nomogram models have previously been constructed to predict distant metastasis and survival of esophageal cancer patients with LM 14, 15, 19, and a population-based study analyzed factors influencing the diagnosis and survival of esophageal cancer patients with LM 16. The primary purpose of this research is to create a nomogram model that can predict the diagnosis and survival of esophageal cancer lung metastases.

We established two nomograms based on the SEER database using logistic regression analysis and cox regression analysis, and then tested their predictive potential using the ROC curve, C-index, calibration curve, and Kaplan-Meier curve. The AUC value and C index are both greater than 0.7, indicating that the model can predict well 20. The calibration curves clearly indicated that the projected survival was very consistent with the actual survival, and all AUC values and C-indices in this investigation were more than 0.7, indicating good predictive power. The findings revealed that the nomograms we produced are capable of accurately predicting the survival time of esophageal cancer lung metastases as well as the frequency of esophageal cancer lung metastases. As a result, our research has significant implications for clinical decision-making.

Age, T stage, pathological type, chemotherapy, radiation, and extrapulmonary metastatic site were identified to be independent predictive variables for esophageal cancer lung metastasis in a recent study by Jida Guo *et al.* 16. This is roughly in line with our findings. Despite several limitations, such as the lack of further nomogram building, this is the first study on early detection and prognostic variables for esophageal cancer lung metastasis. As a result, we conducted this investigation based on their findings. Surgery, bone/brain/liver metastases, radiation, and chemotherapy were revealed to be the critical factors impacting whether esophageal cancer develops lung metastases, with surgery being the most important factor affecting the occurrence of lung metastases in esophageal cancer. However, because surgery alone has a high recurrence rate, chemotherapy or chemoradiotherapy is typically used as an adjuvant to surgery 21, which is consistent with the findings of this study. Chemotherapy, liver metastases, bone metastases, and radiation were also discovered to be the critical factors affecting the prognosis of esophageal cancer lung metastases. Previous research has also identified chemotherapy as a crucial determinant impacting the prognosis of distant metastasis in esophageal cancer 14, and some clinical studies have also found that palliative chemotherapy can improve esophageal cancer patients' survival time and quality of life 22, 23. The current treatment approaches for patients with esophageal cancer distant metastases include primarily radiotherapy, chemotherapy, or chemoradiotherapy 24, which is compatible with our findings.

Radiotherapy, chemotherapy, bone metastases and liver metastases were found to be independent prognostic variables for esophageal cancer lung metastases in multivariate cox regression analysis. Surprisingly, brain metastasis (*P*=0.09) and surgery (*P*=0.527) were not found to be independent predictive variables for esophageal cancer lung metastases. This finding could be related to selection bias, as well as a low rate of brain metastases (6.7%, n=18) and surgery (3.3%, n=9). Despite this, the nomogram produced in this study demonstrated good predictive ability in both the training and validation cohorts, allowing clinicians to make better clinical decisions and allocate medical resources more efficiently.

Few studies have employed PSM in the previous decade to indicate differences in survival between esophageal cancer patients with LM and those without LM, based on the information we have. PSM, a commonly used statistical analysis method, can reduce confounding bias caused by variable correlation and eliminate the potential influence of other variables 25. We used PSM to balance other variables for patients with and without lung metastases and discovered that the median survival time of the control group was strikingly reduced after PSM (18 vs 8 months). Patients with esophageal cancer lung metastases remained shorter than those without lung metastases in the post-PSM cohort (Figure 2). The median OS for esophageal cancer patients with lung metastases is only 5 months, demonstrating the importance of this research.

However, there are several limitations to this research. For starters, it's due to the SEER database itself. Despite the large number of cases in the SEER database, it does not cover the whole American population. Selection bias is unavoidable in the data screening process due to inadequate information in specific areas, such as whether there is liver, lung, bone, or brain metastases. We are unable to undertake a more in-depth analysis since the SEER database lacks information on the progression of esophageal cancer lung metastases, as well as the size and location of metastases. Second, we are unable to do external validation of these two nomograms based on our current data. Third, prospective studies and randomized controlled trials should be used to confirm the nomograms we created.

## Conclusion

The survival time of esophageal cancer patients with lung metastasis was found to be significantly shorter than that of those without lung metastasis, as demonstrated both before and after propensity score matching. Additionally, our study revealed that the risk of esophageal cancer lung metastases can be accurately predicted using a nomogram incorporating various clinical variables. Looking forward, the implications of our findings extend to the clinical setting, particularly in assisting clinicians in making informed treatment decisions for esophageal cancer patients. The predictive model developed in this study serves as a valuable tool in clinical decision-making by providing decision support for treatment planning. Based on the model's results, clinicians can more accurately assess whether patients require surgery, radiotherapy, chemotherapy, or combined therapies, and determine the priority and duration of each treatment modality.

In addition, personalized treatment plans derived from predictive models can provide patients with tailored treatments. For high-risk patients, more aggressive treatment strategies can be chosen, while low-risk patients can benefit from a more conservative treatment approach. This personalized approach not only maximizes treatment efficacy, but also minimizes unwanted side effects and ultimately improves patients' quality of life. Our study highlights the importance of integrating predictive models into clinical practice to optimize treatment decisions for patients with esophageal cancer. Using these models, clinicians can provide more precise and personalized treatment, thereby improving patient outcomes and advancing the field of oncology.

Despite the progress made in this study in exploring predictive models for esophageal cancer lung metastasis, there are still some limitations to consider. First, limitations of the SEER database include incomplete coverage and possible selection bias in data screening. Second, the lack of external validation makes the generalization ability of the two proposed nomograms has not been verified. In addition, further in-depth analyses are limited by the lack of detailed information on tumor metastatic progression in the data. Future prospective studies and randomized controlled trials will be necessary to verify the validity of the model. Nonetheless, this study remains important for clinical decision-making and patient management and provides a useful reference direction for future research.

## Figures and Tables

**Table 1:** Characteristics of patients before and after PSM based on whether or LM

		The pre-PSM cohort					The post-PSM cohort				
		Lung metastasis		Non-lung metastasis			Lung metastasis		Non-lung metastasis		
		N	%	N	%	*P*	N	%	N	%	*P*
		n=400		n=6021			n=393		n=393		
Age	23-66	240	60.00	3632	60.32	0.927	235	59.80	243	61.83	0.076
	67-72	84	21.00	1290	21.43		84	21.37	61	15.52	
	73-80	76	19.00	1099	18.25		74	18.83	89	22.65	
Race	White	307	76.75	5101	84.72	<0.001	305	77.61	305	77.61	0.034
	Black	63	15.75	580	9.63		60	15.27	43	10.94	
	Other	30	7.50	340	5.65		28	7.12	45	11.45	
Gender	Female	52	13.00	1094	18.17	0.008	50	12.72	49	12.47	1
	Male	348	87.00	4927	81.83		343	87.28	344	87.53	
Primary Site	Upper third	19	4.75	281	4.67	0.005	19	4.83	27	6.87	0.297
	Middle third	69	17.25	884	14.68		68	17.30	56	14.25	
	Lower third	236	59.00	4032	66.97		232	59.03	246	62.60	
	Other	76	19.00	824	13.69		74	18.83	64	16.28	
Grade	Grade I	11	2.75	441	7.32	<0.001	11	2.80	13	3.31	0.918
	Grade II	159	39.75	2638	43.81		158	40.20	151	38.42	
	Grade III	227	56.75	2845	47.25		221	56.23	225	57.25	
	Grade IV	3	0.75	97	1.61		3	0.76	4	1.02	
Histologic	Squamous cell carcinoma	147	36.75	1764	29.30	0.005	142	36.13	138	35.11	0.792
	Adenocarcinoma	221	55.25	3791	62.96		219	55.73	227	57.76	
	Other	32	8.00	466	7.74		32	8.14	28	7.12	
T stage	T1	145	36.25	1572	26.11	<0.001	143	36.39	106	26.97	<0.001
	T2	13	3.25	816	13.55		13	3.31	40	10.18	
	T3	102	25.50	2890	48.00		100	25.45	152	38.68	
	T4	140	35.00	743	12.34		137	34.86	95	24.17	
N stage	N0	95	23.75	2314	38.43	<0.001	94	23.92	102	25.95	0.024
	N1	239	59.75	2627	43.63		235	59.80	199	50.64	
	N2	36	9.00	808	13.42		35	8.91	58	14.76	
	N3	30	7.50	272	4.52		29	7.38	34	8.65	
Surgery	No	388	97.00	3277	54.43	<0.001	381	96.95	382	97.20	1
	Yes	12	3.00	2744	45.57		12	3.05	11	2.80	
Radiation	No	214	53.50	1849	30.71	<0.001	210	53.44	212	53.94	0.943
	Yes	186	46.50	4172	69.29		183	46.56	181	46.06	
Chemotherapy	No	148	37.00	1439	23.90	<0.001	142	36.13	123	31.30	0.174
	Yes	252	63.00	4582	76.10		251	63.87	270	68.70	
Bone metastasis	No	304	76.00	5781	96.01	<0.001	303	77.10	306	77.86	0.864
	Yes	96	24.00	240	3.99		90	22.90	87	22.14	
Brain metastasis	No	375	93.75	5960	98.99	<0.001	371	94.40	373	94.91	0.874
	Yes	25	6.25	61	1.01		22	5.60	20	5.09	
Liver metastasis	No	235	58.75	5566	92.44	<0.001	234	59.54	240	61.07	0.716
	Yes	165	41.25	455	7.56		159	40.46	153	38.93	
Tumor size	1--25	38	9.50	1360	22.59	<0.001	38	9.67	34	8.65	0.877
	26-63	200	50.00	3153	52.37		197	50.13	201	51.15	
	64-560	162	40.50	1508	25.05		158	40.20	158	40.20	

We used the x-tile v3.6.1 (Yale University) to determine the optimal cutoffs for tumor size and age. PSM: propensity score matching

**Table 2:** Univariate and multivariate logistic regression for analyzing associated factors for developing LM

		Univariate		Multivariate	
		HR (95% *CI*)	*P*	HR (95% *CI*)	*P*
Age	23-66	1			
	67-72	0.985 (0.763-1.274)	0.911		
	73-80	1.047 (0.802-1.366)	0.738		
Race	White	1		1	
	Black	1.805 (1.358-2.398)	<0.001	1.209 (0.854-1.710)	0.284
	Other	1.466 (0.992-2.167)	0.055	1.240 (0.794-1.937)	0.345
Sex	Female	1		1	
	Male	1.486 (1.103-2.003)	0.009	1.492 (1.074-2.073)	0.017
Primary Site	Upper third	1		1	
	Middle third	1.154 (0.683-1.952)	0.592	1.411 (0.804-2.476)	0.231
	Lower third	0.866 (0.534-1.403)	0.588	1.270 (0.730-2.211)	0.398
	Other	1.364 (0.810-2.296)	0.242	1.395 (0.792-2.456)	0.249
Grade	Well differentiated; Grade I	1		1	
	Moderately differentiated; Grade II	2.416 (1.301-4.489)	0.005	1.836 (0.950-3.549)	0.071
	Poorly differentiated; Grade III	3.199 (1.732-5.907)	<0.001	1.836 (0.953-3.539)	0.069
	Undifferentiated; anaplastic; Grade IV	1.240 (0.339-4.529)	0.745	0.649 (0.163-2.590)	0.541
Histologic	Squamous cell carcinoma	1		1	
	Adenocarcinoma	0.700 (0.564-0.868)	0.001	0.777 (0.569-1.061)	0.113
	Other	0.824 (0.555-1.224)	0.338	0.669 (0.414-1.081)	0.101
T stage	T1	1		1	
	T2	0.173 (0.097-0.307)	<0.001	0.279 (0.153-0.507)	<0.001
	T3	0.383 (0.295-0.497)	<0.001	0.576 (0.426-0.779)	<0.001
	T4	2.043 (1.594-2.618)	<0.001	1.209 (0.911-1.604)	0.189
N stage	N0	1		1	
	N1	2.216 (1.736-2.829)	<0.001	1.634 (1.241-2.151)	<0.001
	N2	1.085 (0.733-1.606)	0.682	1.062 (0.688-1.639)	0.786
	N3	2.687 (1.749-4.127)	<0.001	1.807 (1.098-2.975)	0.020
Surgery	No	1		1	
	Yes	0.037 (0.021-0.066)	<0.001	0.083 (0.045-0.151)	<0.001
Radiation	No	1		1	
	Yes	0.385 (0.314-0.472)	<0.001	0.621 (0.482-0.801)	<0.001
Chemotherapy	No	1		1	
	Yes	0.535 (0.433-0.661)	<0.001	0.641 (0.496-0.828)	0.001
Bone metastasis	No	1		1	
	Yes	7.607 (5.846-9.898)	<0.001	2.806 (2.093-3.762)	<0.001
Liver metastasis	No	1		1	
	Yes	8.589 (6.887-10.711)	<0.001	3.085 (2.378-4.002)	<0.001
Brain metastasis	No	1		1	
	Yes	6.514 (4.043-10.495)	<0.001	2.602 (1.499-4.516)	0.001
Tumor size	1-25	1		1	
	26-63	2.270 (1.596-3.230)	<0.001	1.260 (0.853-1.862)	0.246
	64-560	3.845 (2.680-5.515)	<0.001	1.455 (0.971-2.180)	0.069

HR: hazard ratio; CI: confidence interval

**Table 3:** Statistical characteristics of patients with esophageal cancer lung metastases

		Training cohort (N=268)		Validation cohort (N=132)		
		n	%	n	%	*P*
Age	23-66	161	60.07	79	59.85	0.827
	67-72	58	21.64	26	19.70	
	73-80	49	18.28	27	20.45	
Race	White	204	76.12	103	78.03	0.872
	Black	44	16.42	19	14.39	
	Other	20	7.46	10	7.58	
Sex	Female	31	11.57	21	15.91	0.268
	Male	237	88.43	111	84.09	
Primary Site	Upper third	14	5.22	5	3.79	0.172
	Middle third	47	17.54	22	16.67	
	Lower third	149	55.60	87	65.91	
	Other	58	21.64	18	13.64	
Grade	Well differentiated; Grade I	5	1.87	6	4.55	0.443
	Moderately differentiated; Grade II	105	39.18	54	40.91	
	Poorly differentiated; Grade III	156	58.21	71	53.79	
	Undifferentiated; grade iv	2	0.75	1	0.76	
Histologic	Squamous cell carcinoma	98	36.57	49	37.12	0.086
	Adenocarcinoma	143	53.36	78	59.09	
	Other	27	10.07	5	3.79	
T stage	T1	99	36.94	46	34.85	0.097
	T2	8	2.99	5	3.79	
	T3	59	22.01	43	32.58	
	T4	102	38.06	38	28.79	
N stage	N0	60	22.39	35	26.52	0.731
	N1	161	60.07	78	59.09	
	N2	25	9.33	11	8.33	
	N3	22	8.21	8	6.06	
Surgery	No	259	96.64	129	97.73	0.758
	Yes	9	3.36	3	2.27	
Radiation	No	156	58.21	58	43.94	0.008
	Yes	112	41.79	74	56.06	
Chemotherapy	No	105	39.18	43	32.58	0.226
	Yes	163	60.82	89	67.42	
Bone metastasis	No	202	75.37	102	77.27	0.710
	Yes	66	24.63	30	22.73	
Liver metastasis	No	150	55.97	85	64.39	0.130
	Yes	118	44.03	47	35.61	
Brain metastasis	No	250	93.28	125	94.70	0.665
	Yes	18	6.72	7	5.30	
Tumor size	1-25	24	8.96	14	10.61	0.855
	26-63	134	50.00	66	50.00	
	64-560	110	41.04	52	39.39	

We divided 400 patients with LM into training (2012-2015, n=268) and internal validation (2010-2011, n=132) cohorts by year of diagnosis. No statistically significant differences were found for most variables.

**Table 4:** Univariate and multivariate Cox proportional hazards regression analysis in esophageal cancer patients with lung metastasis

		OS			
		Univariate		Multivariate	
		HR (95% *CI*)	*P*	HR (95% *CI*)	*P*
Age	23-66	1			
	67-72	0.868(0.635-1.186)	0.374		
	73-80	1.319(0.950-1.832)	0.098		
Race	White	1		1	
	Black	1.479(1.051-2.082)	0.025	1.490 (0.995-2.232)	0.053
	Other	1.064(0.656-1.727)	0.801	1.057 (0.614-1.820)	0.840
Sex	Female	1			
	Male	0.856(0.586-1.248)	0.418		
Primary Site	Upper third	1			
	Middle third	1.186(0.623-2.259)	0.604		
	Lower third	1.471(0.813-2.660)	0.202		
	Other	1.223(0.652-2.292)	0.530		
Grade	Well differentiated; Grade I	1		1	
	Moderately differentiated; Grade II	0.388(0.156-0.963)	0.041	0.931 (0.360-2.406)	0.882
	Poorly differentiated; Grade III	0.518(0.211-1.271)	0.151	1.017 (0.399-2.593)	0.972
	Undifferentiated; grade iv	2.735(0.525-14.251)	0.232	5.032 (0.877-28.887)	0.070
Histologic	Squamous cell carcinoma	1		1	
	Adenocarcinoma	0.925(0.707-1.211)	0.573	1.070 (0.767-1.494)	0.690
	Other	1.542(0.994-2.391)	0.053	1.499 (0.907-2.476)	0.114
T stage	T1	1		1	
	T2	0.543(0.237-1.242)	0.148	0.792 (0.342-1.835)	0.586
	T3	0.669(0.476-0.940)	0.021	0.929 (0.646-1.334)	0.689
	T4	1.056(0.795-1.403)	0.707	1.195 (0.887-1.610)	0.242
N stage	N0	1			
	N1	0.692(0.510-0.940)	0.018		
	N2	0.909(0.561-1.472)	0.698		
	N3	0.801(0.481-1.333)	0.393		
Surgery	No	1		1	
	Yes	0.426(0.199-0.908)	0.027	0.775 (0.351-1.708)	0.527
Radiation	No	1		1	
	Yes	0.745(0.578-0.959)	0.022	0.750 (0.565-0.997)	0.048
Chemotherapy	No	1		1	
	Yes	0.255(0.192-0.339)	<0.001	0.278 (0.206-0.376)	<0.001
Bone metastasis	No	1		1	
	Yes	1.327(0.996-1.768)	0.053	1.449(1.072-1.960)	0.016
Liver metastasis	No	1		1	
	Yes	1.587(1.235-2.041)	<0.001	1.429(1.092-1.869)	0.009
Brain metastasis	No	1		1	
	Yes	1.626(1.003-2.634)	0.049	1.579(0.931-2.677)	0.090
Tumor size	1-25	1			
	26-63	0.858(0.549-1.343)	0.503		
	64-560	1.141(0.723-1.799)	0.571		

We included factors with* P*<0.10 in univariate cox regression into the multivariate analysis.
